# Topical anti-inflammatory activity of *Polygonum cuspidatum *extract in the TPA model of mouse ear inflammation

**DOI:** 10.1186/1476-9255-5-1

**Published:** 2008-02-08

**Authors:** Eve E Bralley, Phillip Greenspan, James L Hargrove, Louise Wicker, Diane K Hartle

**Affiliations:** 1 Department of Pharmaceutical and Biomedical Sciences, Nutraceutical Research Laboratories, University of Georgia, Athens, GA, USA; 2 Department of Food and Nutrition, Nutraceutical Research Laboratories, University of Georgia, Athens, GA, USA; 3 Department of Food Science and Technology, University of Georgia, Athens, GA, USA

## Abstract

**Background:**

This study tested the ability of a characterized extract of *Polygonum cuspidatum *(PCE) to inhibit mouse ear inflammation in response to topical application of 12-*O-*tetradecanoylphorbol-13-acetate (TPA).

**Methods:**

A 50% (wt:vol) ethanolic solution of commercial 200:1 PCE was applied to both ears of female Swiss mice (n = 8) at 0.075, 0.15, 0.3, 1.25 and 2.5 mg/ear 30 min after TPA administration (2 μg/ear). For comparison, 3 other groups were treated with TPA and either 1) the vehicle (50% ethanol) alone, 2) indomethacin (0.5 mg/ear), or 3) *trans*-resveratrol (0.62 mg/ear). Ear thickness was measured before TPA and at 4 and 24 h post-TPA administration to assess ear edema. Ear punch biopsies were collected at 24 h and weighed as a second index of edema. Myeloperoxidase activity was measured in each ear punch biopsy to assess neutrophil infiltration.

**Results:**

PCE treatment at all doses significantly reduced ear edema compared to the TPA control. The PCE response was dose-dependent and 2.5 mg PCE significantly inhibited all markers of inflammation to a greater extent than indomethacin (0.5 mg). MPO activity was inhibited at PCE doses ≥ 1.25 mg/ear. *Trans-*resveratrol inhibited inflammation at comparable doses.

**Conclusion:**

PCE inhibits development of edema and neutrophil infiltration in the TPA-treated mouse ear model of topical inflammation.

## Background

*Polygonum cuspidatum *Sieb et Zucc., commonly called Japanese knotweed or Mexican bamboo, is a member of the Polygonaceae family that is widely distributed in Asia and North America. Interest in *Polygonum cuspidatum *(PC) has increased owing to the high concentration of resveratrol and its glycosides in the root [1,2]. In traditional Chinese medicine, PC is called *Hu Zhang *and is used as an analgesic, antipyretic, diuretic, and an expectorant. Traditional uses include treatments for arthralgia, chronic bronchitis, jaundice, amenorrhea, and high blood pressure [3]. Several studies have evaluated the antioxidant capacity of *Polygonum cuspidatum *extract (PCE) [4,5], and anti-inflammatory activities such as inhibition of NF-kB have been reported [6-8]. At present, studies of PCE effects on classic symptoms of inflammation such as edema and neutrophil infiltration are lacking.

PCE is used as an ingredient in many nutraceutical product formulations because of its high concentration of *trans*-resveratrol, a polyphenolic *trans*-stilbene (3, 4'-5-trihydroxystilbene). Resveratrol and related phytochemicals produce antioxidant, cardioprotective, immunomodulatory, chemopreventive, anti-bacterial, anti-fungal, and anti-viral effects [9-14]. The concentration of resveratrol in sources such as grapes and red wine varies depending on environmental conditions [15]. Therefore, PCE is being used commercially as an additive to standardize resveratrol concentration in extracts of grape pomace (skins and seeds) that have low or variable natural concentrations [16,17]. Also, additive and synergistic effects have been noted for combinations of resveratrol and flavonoids such as quercetin and ellagic acid [18].

PCE has not been tested in the tetradecanoylphorbol acetate (TPA)-treated mouse ear model of inflammation. This model evaluates whether pharmaceutical agents or natural products may block the inflammatory response to topical TPA [19-21]. Because PCE is being used as an ingredient in cosmeceutical products that are applied to the skin and in nutraceutical products that are ingested, it is worthwhile to test PCE activity in this model. The skin and gastrointestinal mucosa are both subject to inflammation, but it is far easier to screen for anti-inflammatory effects on an accessible surface than on an internal epithelium. Therefore, the present study tested whether PCE has topical anti-inflammatory activities in the well-characterized TPA-induced mouse ear model of inflammation, edema, and PMN leukocyte infiltration [22,23]. Total phenolics and ferric reducing antioxidant power (FRAP values) were measured in the ethanolic PCE because both characteristics may reflect the degree of anti-inflammatory activity of the preparation. For example, Chung et. al. reported that edema formation in the TPA model may be regulated by H_2_O_2 _generation [24], as evidenced by anti-inflammatory activity of several antioxidant compounds [25,26].

## Materials and methods

### Materials

12-*O*-Tetradecanoylphorbol 13-acetate, hexadecyltrimethylammonium bromide, indomethacin (minimum 99% TLC), 3,3',5,5'-tetramethylbenzidine dihydrochloride, *N, N-*dimethylformamide, trans-3,4',5-trihydroxystilbene (*trans-*resveratrol), Folin-Ciocalteu reagent, gallic acid, and 10 mM 2,4,6-tripyridyl]-1,3,5-triazine (TPTZ) were all purchased from Sigma-Aldrich Chemical Co. (St. Louis, MO). *Polygonum cuspidatum *200:1 powdered extract was purchased from Supplemental Health Formulations (Mayer, AZ).

### Preparation of ethanolic solution of Polygonum cuspidatum extract (PCE)

PCE used in this study was a 200-fold concentrate prepared from PC root grown in China. Chemical analysis from Supplemental Health Formulations reported that the *trans-*resveratrol complex was at least 500 mg/g and emodin content was < 20 mg/g. The 200:1 PC powder was dissolved in 50% ethanol (1 part PC to 9 parts ethanol) and stirred for 1 h at 23°C. The mixture was centrifuged (1500 rpm for 10 min, 4°C) and the supernatant was diluted for topical dose-response applications in this study. The majority of the powder was not soluble in 50% ethanol under these conditions.

### Chromatographic analysis of the Polygonum cuspidatum ethanolic solution

The ethanolic extract was diluted 400-fold and subjected to HPLC analysis using an ESA (Chelmsford, MA) system consisting of a Model 582 Solvent Delivery Module, a Model 542 autosampler maintained at 6°C and a Model 5600A CoulArray detector at 250 mV. The column was an MCM C18 (4.6 × 150 mm, 5–120 A) from MC Medical, Japan. Mobile phase A was 75 mM citric acid, 25 mM ammonium acetate and 10% acetonitrile; Mobile phase B was similar to A but with 50% acetonitrile. The gradient was linear from 0–17 minutes from 10%A to 80%B. Flow rate was 1.0 ml/min and 20 μl of sample was injected. Resveratrol eluted between 16.2 and 16.8 minutes as judged by a standard obtained from Sigma-Aldrich (St. Louis, MO).

### Measurement of total phenolic compounds

Total phenolic acid content of each extract was measured by the method of Slinkard and Singleton [27] with minor modifications. Triplicate samples of a 1:10 extract (wt/vol) (20 μL) were added to 1.58 mL of distilled water in 3 mL polystyrene cuvettes. 100 μL of Folin-Ciocalteu reagent was added and the sample was mixed well. Within 10 minutes, 300 μL of sodium carbonate solution (200 g Na_2_CO_3 _in 1 L distilled water) was added. Solutions were incubated for 2 h at room temperature. Absorbance was measured at 765 nm. Total phenolic acid concentration was calculated from a gallic acid standard curve (0–500 mg/L) and expressed as gallic acid equivalents per gram 200:1 PCE powder.

### Measurement of FRAP values (Ferric Reducing Antioxidant Power)

The antioxidant activity of a 1:10 (wt/vol) extraction was determined in triplicate by the FRAP method [28]. 10 μL of the sample or standard, 30 μL of distilled water and 300 μL of FRAP reagent were mixed. FRAP reagent was made by mixing 25 mL acetate buffer (300 mM, pH 3.6), 2.5 mL of 10 mM TPTZ solution dissolved in 40 mM HCl, and 2.5 mL of 20 mM ferric chloride solution. The solutions were incubated at 37°C for six minutes then 340 μL of distilled water was added. The absorbance of the sample or standards was read immediately at 593 nm. FRAP value was calculated from a standard curve of ferrous sulfate (0–1 mmol/L) and the antioxidant power of the PCE was expressed as mmol ferrous sulfate equivalents/100 g dry weight of the 200:1 PCE powder.

### Animals

All animal experiments were approved by the Institutional Animal Care and Use Committee (IACUC) at the University of Georgia and conducted according to IACUC guidelines. The sample size of 8 animals for each test group was justified on the basis of a pilot experiment showing that the sample standard deviation (s) for measurements of ear edema was about 5% of the measured value and the average expected difference (d) between TPA treated ears and PCE-treated ears was about 0.2 mm. Assuming that α = 0.05 and 1 - β = 0.9, the formula used was n (sample size) = 1 + 21*(s/d)^2 ^[29]. The formula gave 6.25, which was increased to 8 in case of unexpected experimental problems. Female Swiss Webster mice (Harlan Laboratories, Indianapolis, IN) weighing 22–25 g were housed in groups of 4 in large shoebox cages. All groups were fed a standard rodent diet (TestDiet^® ^570B, Purina Mills, St. Louis, MO) *ad libitum *with free access to water. Animals were in the fed condition throughout the experiment. Photoperiods equaled 12 h of light and 12 h of darkness daily, with the environmental temperature maintained at 21°C.

### TPA-induced mouse ear edema

Edema was induced in both ears of each mouse by the topical application of 2 μg TPA dissolved in 20 μL of acetone to both the inner and outer ear surfaces. Thirty minutes after the application of TPA, the inner and outer surface of each ear was treated (10 μL to each side) with 50% ethanolic solutions of PCE in doses of 0.075, 0.15, 0.3, 1.25 and 2.5 mg PCE/ear (n = 8 at each dosage). Comparisons included equal volumes of 50% ethanol (vehicle control), indomethacin (0.5 mg/ear dissolved in 50% ethanol as an anti-inflammatory drug standard), or a 50% ethanol solution of trans-3, 5, 4'-trihydroxystilbene (resveratrol, 0.6 mg/ear). The thickness of each ear was measured using a micrometer (Mitutoyo Series IP65, Mitutoyo America, Aurora, IL) before and at 4 h and 24 h after TPA administration. The micrometer was applied near the top of the ear distal to the cartilaginous ridges. At 24 h each animal was sacrificed with CO_2 _inhalation by the IACUC approved protocol. Ear punch biopsies (6 mm diameter hole punch) were taken immediately, weighed, frozen and stored at -80°C. A single investigator performed all ear measurements and biopsies in order to standardize the procedure and reduce experimental error.

### Myeloperoxidase assay

Tissue MPO (MPO, E.C. 1.11.1.7) activity was measured in biopsies taken from both ears 24 h after TPA administration using a method by Suzuki et. al. [30] and modified by De Young et. al. [31]. Each mouse ear biopsy was placed in 0.75 mL of 80 mM phosphate-buffered saline (PBS) pH 5.4 containing 0.5% hexadecyltrimethyl-ammonium bromide (HTAB). Each sample was homogenized for 45 s at 4°C with a small sample laboratory Tissue Tearor Homogenizer Model 985-370 (Biospec Products, Bartlesville, OK). The homogenate was transferred quantitatively to a microcentrifuge tube with an additional 0.75 mL HTAB in PBS. The 1.5 mL sample was centrifuged at 12,000 × g for 15 min, maintained at 4°C. Triplicate 30 μL samples of the resulting supernatant were added to 96-well microtiter plate wells. For the MPO assay, 200 μL of a mixture containing 100 μL of 80 mM PBS (pH 5.4), 85 μL of 0.22 M PBS (pH 5.4), and 15 μL of 0.017% hydrogen peroxide were added to each well. 20 μL of 18.4 mM tetramethylbenzidine HCl in 8% aqueous dimethylformamide was added to start the reaction. Microtiter plates were incubated at 37°C for 3 min, and then placed on ice. The reaction was stopped with the addition of 30 μL of 1.46 M sodium acetate, pH 3.0. MPO enzyme activity was assessed colorimetrically using a BioTek Microplate Reader (Winooski, VT) at an absorbance wavelength of 630 nm. MPO activity was expressed as optical density (OD)/biopsy.

### Statistical analysis

Data are expressed as the mean ± standard error of the mean (SEM). Statistical evaluations used t-tests and one-way analysis of variance (ANOVA) with post-hoc tests for significance of differences by the Student-Newman-Keuls Method. Statistical significance was considered at p < 0.05.

## Results

### Total phenolics and FRAP values in PCE

**Figure 1:**
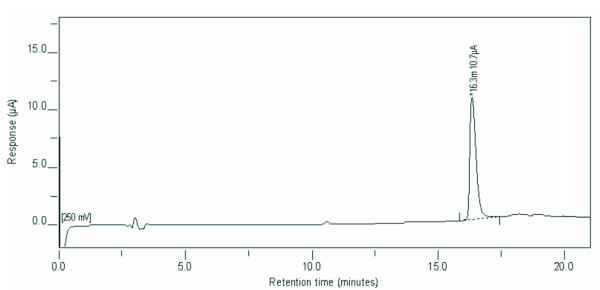
**Chromatogram of ethanol-soluble PCE fraction**. A single major peak was observed in the chromatogram of the 50% ethanol soluble fraction of PCE that eluted at 16.3 min with the same retention time as authentic *trans*-resveratrol (not shown).

A 50% ethanolic extract (1:10 wt/vol) of the 200:1 PCE yielded 188 mg of total phenolics (gallic acid equivalents) per gram of PCE. Antioxidant power based on the FRAP assay was 85 mmol ferrous sulfate equivalents/100 g dry weight of PCE. Most of the solids in the commercial extract were not soluble in 50% ethanol. The dry weight of the ethanol-insoluble pellet remaining after centrifugation of the extract from 1.0 g of powder equaled 0.76 g, indicating that the majority was not soluble in 50% ethanol. Chromatography of the ethanol-soluble material as described in the Methods section showed only one major peak, which co-eluted with authentic trans-resveratrol (Figure 1).

### Ear edema

**Figure 2:**
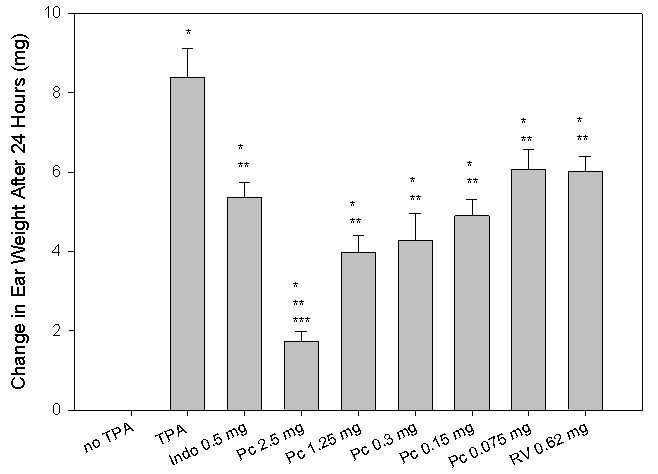
**Change in ear thickness 4 and 24 h after TPA application**. Ear thickness was measured with a digital micrometer 4 and 24 h after application of 2 μg TPA. Abbreviations include Indo (indomethacin), PCE (*Polygonum cuspidatum *extract), and RV (resveratrol). Results represent means ± SEM. *p ≤ 0.05 compared to no TPA, **p ≤ 0.05 compared to TPA control, ***p ≤ 0.05 compared to indomethacin (Indo).

Ear edema was observed in all TPA-treated animals by 4 h and 24 h after treatment. In animals treated only with vehicle (50% ethanol), initial ear thickness equaled 0.27 ± 0.01 mm (mean ± SEM). Ear thickness increased to 0.42 ± 0.01 mm at 4 h and 0.46 ± 0.02 mm by 24 h after TPA treatment. PCE-treated experimental groups showed significantly reduced ear edema compared to TPA treatment alone. Dosages tested included 0.075, 0.15, 0.3, 1.25 and 2.5 mg PCE/ear (n = 8 at each dosage). PCE at 2.5, 1.25, and 0.3 mg per ear was as effective as indomethacin (0.5 mg/ear) in reducing edema (Figure 2). These treatments inhibited edema 61%, 55%, 52%, and 65% (Indo), respectively compared to TPA treated with vehicle controls. In comparison, 0.62 mg of commercially purified *trans-*resveratrol inhibited edema by only 35%. At 24 h, all experimental groups had significantly reduced ear edema compared to TPA alone except PCE at 0.075 mg per ear and the *trans-*resveratrol-treated groups. PCE at 1.25, 0.3, and 0.15 mg per ear inhibited edema as well as indomethacin (58%, 36%, 40%, respectively, vs 45% for indomethacin. PCE applied at 2.5 mg per ear was significantly more effective than indomethacin in reducing edema with a 73% reduction compared to the TPA treated vehicle control.

Edema was also indicated by changes in ear punch masses at 24 h, and the treatment effects were similar to the changes in ear thickness shown in Figure 2. Typical masses of ear punch biopsies at 24 h were 9.1 ± 0.3 mg in vehicle-treated controls compared to 17.5 ± 0.7 mg in TPA-treated animals. Ear punch biopsy weights were significantly lower in all PCE groups compared to the TPA-treated control group (data not shown). For example, 2.5 mg of PCE reduced the change in ear mass to 1.3 ± 0.25 mg (an 80% reduction), which was significantly greater than the reduction by 0.5 mg of indomethacin to 5.36 ± 0.39 mg (36% reduction). Resveratrol (0.62 mg) produced an effect similar to indomethacin, and reduced the change in ear thickness to 6.02 ± 0.38 mg.

### Myeloperoxidase activity

**Figure 3:**
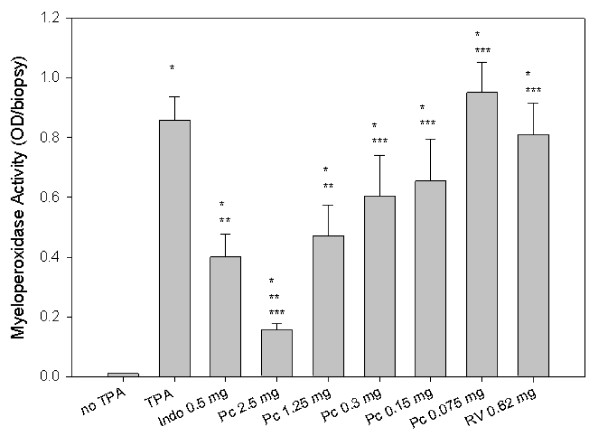
**Myeloperoxidase activity**. Myeloperoxidase activity (an index of neutrophil activation) was measured in ear punches 24 h after TPA administration. Abbreviations include Indo (indomethacin), PCE (*Polygonum cuspidatum *extract), and RV (resveratrol). Results represent means ± SEM. *p ≤ 0.05 compared to no TPA, **p ≤ 0.05 compared to TPA control, ***p ≤ 0.05 compared to indomethacin.

Myeloperoxidase activity was measured in the ear punch biopsies taken 24 h after TPA administration as an index of neutrophil infiltration (Figure 3). Biopsies from ears treated with indomethacin at 0.5 mg/ear and PCE at 1.25 and 2.5 mg/ear doses had significantly reduced MPO activity. The higher PCE dose (2.5 mg/ear) decreased MPO to 18% of the activity of the TPA-treated vehicle control group and was significantly more effective at decreasing MPO activity than indomethacin. Indomethacin (0.5 mg/ear) and PCE (1.25 mg/ear) inhibited MPO to the same extent at 53% and 45%, respectively.

## Discussion

An early hallmark of skin irritation and local inflammation in the TPA model is thickening within 1–4 h due to increased vascular permeability, edema and swelling within the dermis [32]. Topical application of PCE significantly inhibited ear edema at 4 h and 24 h after TPA treatment. Secondarily, PMN leukocytes migrate to the dermis within about 24 h and may be estimated by the MPO assay. Both of these inflammatory processes were blocked by topical application of PCE in a dose-dependent manner. PCE at a dose of 2.5 mg/ear reduced edema and inhibited leukocyte infiltration to a greater extent than indomethacin (0.5 mg/ear). Indomethacin is a potent non-steroidal, anti-inflammatory drug. It has an LD_50 _of 50 mg/kg in mice based on a 14 day mortality response [33]. This LD_50_translates to 1.25 mg indomethacin per 25 g mouse, just above the dose administered topically (1 mg/mouse). In contrast, no significant toxicity has been shown for PCE in this bioequivalence range. These data show that an ethanolic solution of PCE reduces inflammation to a similar extent as indomethacin or *trans*-resveratrol.

PCE is widely used in nutraceutical products because of consistently high concentration of resveratrol and its glucosides. Resveratrol derivatives in extracts of PC root include several glycosides [1,34,35]. In addition, PC contains emodin and a glycoside. However, Figure 1 shows that the ethanol-soluble fraction of the commercial concentrate used here was less complex than crude extracts of PC root [2,34,35]. The chromatogram agrees with the certificate of analysis of that PCE powder, 200:1, contains at least 50% *trans-*resveratrol and less than 2% emodin. In our tests, PCE was similar in activity to *trans-*resveratrol on a mass basis (Figures 2 and 3).

Our data are consistent with findings that *trans*-resveratrol and its derivatives have anti-inflammatory activity. For example, resveratrol and its glycosides inhibit human TNF-α and LPS-induced activation of NF-κB [36,37]. Resveratrol inhibits induced production of prostaglandin E_2 _release from human peripheral blood leukocytes [38]. In a model of early colonic inflammation in rats, resveratrol significantly decreases elevated plasma levels of prostaglandin D_2 _and the expression of COX-2 [39]. Resveratrol also inhibits the TPA-induced mouse dorsal skin inflammatory response by reducing NF-κB and activator protein-1 [40,41].

The TPA model of ear inflammation is useful for screening prospective topical anti-inflammatory compounds or botanical extracts that act at a variety of levels. In epidermal cell culture, TPA stimulates cell proliferation and increases the formation of leukotrienes and prostaglandins [42]. Phospholipase A_2 _inhibitors have proven effective against both leukocyte infiltration and edema in the TPA model of ear inflammation [43]. Products of arachidonic acid metabolism such as PGI_2 _and LTB_4 _increase vascular permeability leading to edema during the inflammatory response [23], and compounds inhibiting COX and LOX enzymes have been shown to inhibit TPA-induced inflammation [23]. TPA applied topically to mouse ears promotes mast cell infiltration with release of mediators that increase vascular permeability and promote neutrophil influx [22].

In addition to 50% resveratrol, PCE extract contains compounds such as quercetin and emodin that have anti-inflammatory activities. It is known that additive and syngergistic interactions of polyphenols occur *in vitro *[44,45]. For example, in human leukemia cells, ellagic acid and quercetin interact synergistically with resveratrol to induce apotosis and cell cycle arrest [18]. Emodin, an anthraquinone, is present in PC rhizomes at concentrations similar to resveratrol and piceid [2]. However, the emodin content in PCE is reduced during processing to achieve a final content of ≤ 20 mg/g. This is important because PCE is a constituent in products that are ingested, and it is desirable to reduce the risk of unpleasant gastrointestinal side effects in humans [46]. Emodin is a phytoestrogen with anti-viral and anti-inflammatory actions [47]. It inhibits NF-κB activation and IκB degradation, and decreases gene expression of cell surface adhesion proteins in vascular endothelial cells [6]. Emodin also effectively inhibits gene expression for TNF-α, iNOS, and IL-10 in RAW 264.7 macrophages by activating IκB [48]. Thus, even though emodin levels in PCE have been reduced from levels in crude extracts, it may contribute to the topical anti-inflammatory activity of PCE. The present work shows that PCE and *trans*-resveratrol are anti-inflammatory in the mouse ear model, and that PCE could provide anti-inflammatory properties to cosmeceutical and dermatological products.

## Abbreviations used

TPA: 12-*O-*tetradecanoylphorbol-13-acetate, PMN: Polymorphonuclear, MPO: Myeloperoxidase, TNF-α: Tumor Necrosis Factor – alpha, IL-6, -1β, -8: Interleukin-6, -1β, -8, COX: Cyclooxygenase, LOX: Lipoxygenase, Indo: Indomethacin, PC: *Polygonum cuspidatum*, PCE: *Polygonum cuspidatum *extract in 50% ethanol

## Competing interests

The author(s) declare that they have no competing interests.

## Authors' contributions

The study was conceived by DKH and LW. EB conducted the study as part of her doctoral research under the direction of PG, DKH and JLH. EB and JLH prepared the figures and manuscript, which was reviewed and approved by each of the coauthors.
